# Deep transcranial magnetic stimulation in the treatment of OCD: Comparison of treatment with Double-Cone and H7 coil

**DOI:** 10.1192/j.eurpsy.2026.10715

**Published:** 2026-07-31

**Authors:** M. Jakubec, O. Laskov, N.-A. Böhmová, N. Biačková, A. Čechová, T. Novák, M. Klírová

**Affiliations:** 1 Non-Invasive Brain Stimulation Working Group, National Institute of Mental Health, Klecany; 2 Third Faculty of Medicine, Charles University, Prague, Czech Republic

## Abstract

**Introduction:**

**Obsessive–compulsive disorder (OCD)** is a chronic neuropsychiatric illness characterized by intrusive thoughts (obsessions) and repetitive acts (compulsions). Hyperactivity within the **medial prefrontal cortex (mPFC)** and **anterior cingulate cortex (ACC)**, structures in the cortico-striato-thalamo-cortical (CSTC) loop that affect **inhibitory control**, has been implicated in its pathophysiology (Zhang et al. Front Psychiatry 2019; 10 522). **Deep transcranial magnetic stimulation (dTMS)** is a modern variation on non-invasive brain-stimulation (NIBS) that, unlike other NIBS approaches, **targets deeper cortical structures** to alleviate OCD symptomatology (Laskov & Klírová Neurosci Lett 2021; 755 135906). Its efficacy has been proven in several RCTs and meta-analyses (Trevizol et al. J ECT 2016; 32 262-266; Cruz et al. Psychiatry Res 2022; 317 114869; Carmi et al. Am J Psychiatry 2019; 176 931-938).

**Objectives:**

Two dTMS coils cleared by the US FDA for OCD are available **–** The BrainsWay **H7 coil** and the MagVenture Cool D-B80 **Double-Cone coil**. The Double-Cone coil, approved in 2020 for adjunctive treatment, is electromagnetically less potent in OCD-associated brain structures stimulation than H7 (Tzirini et al. PLoS One 2022; 17 e0263145). We **compared the clinical performance of these two coils** in a preliminary analysis of an ongoing randomised, sham (placebo)-controlled study designed to identify neurobiological predictors of dTMS response.

**Methods:**

**Twenty** DSM-5-diagnosed **OCD patients** underwent six weeks of dTMS. **Ten** received the **Double-Cone stimulation** [active n = 8, sham n = 2] and **ten** the **H7 stimulation** [active n = 8, sham n = 2] (** Figure 1**). Treatment response was defined as ≥ 35% reduction on the Yale–Brown Obsessive Compulsive Scale (Y-BOCS).

**Results:**

The **highest responder rate** occurred with **active H7 stimulation** (4/8; **50%**), followed by active Double-Cone stimulation (1/8; 12.5%); no sham-treated patient responded (** Figure 2**). χ² tests showed no significant differences between coils (χ² = 2.50, p = 0.114) or between active and sham stimulation (χ² = 2.78, p = 0.095). **Mean reduction in Y-BOCS** (indicates improvement) was greatest in **H7-active** (**19.5%**), followed by H7-sham (16.2%), Double-Cone-sham (12.2%), and Double-Cone-active (4.7%). **Figure 3** shows boxplots of percentage Y-BOCS reduction by study group. Logistic regression identified **shorter OCD duration** as a significant **predictor of response** (B = −0.12, p = 0.048). **Younger age** also favoured response (p = 0.071). Although type of stimulation and coil both favoured the H7 coil, these effects did not reach significance (p = 0.086 and p = 0.059, respectively).

**Image 1:**
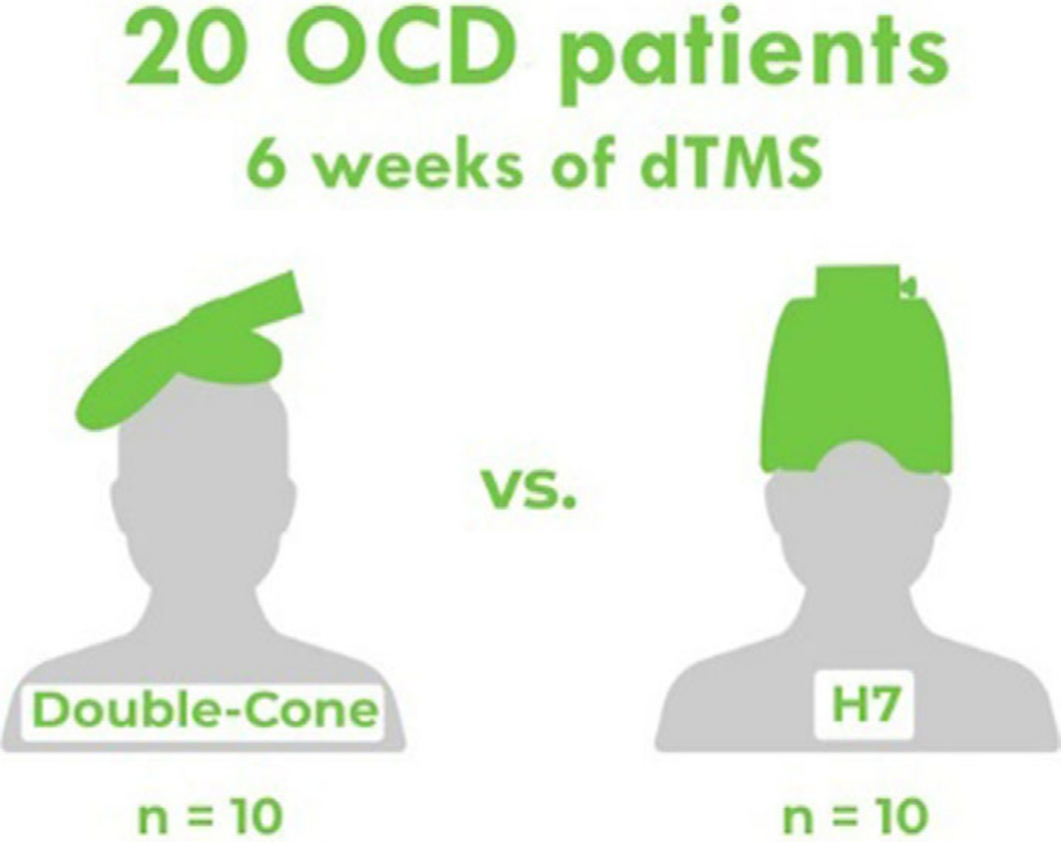

**Image 2:**
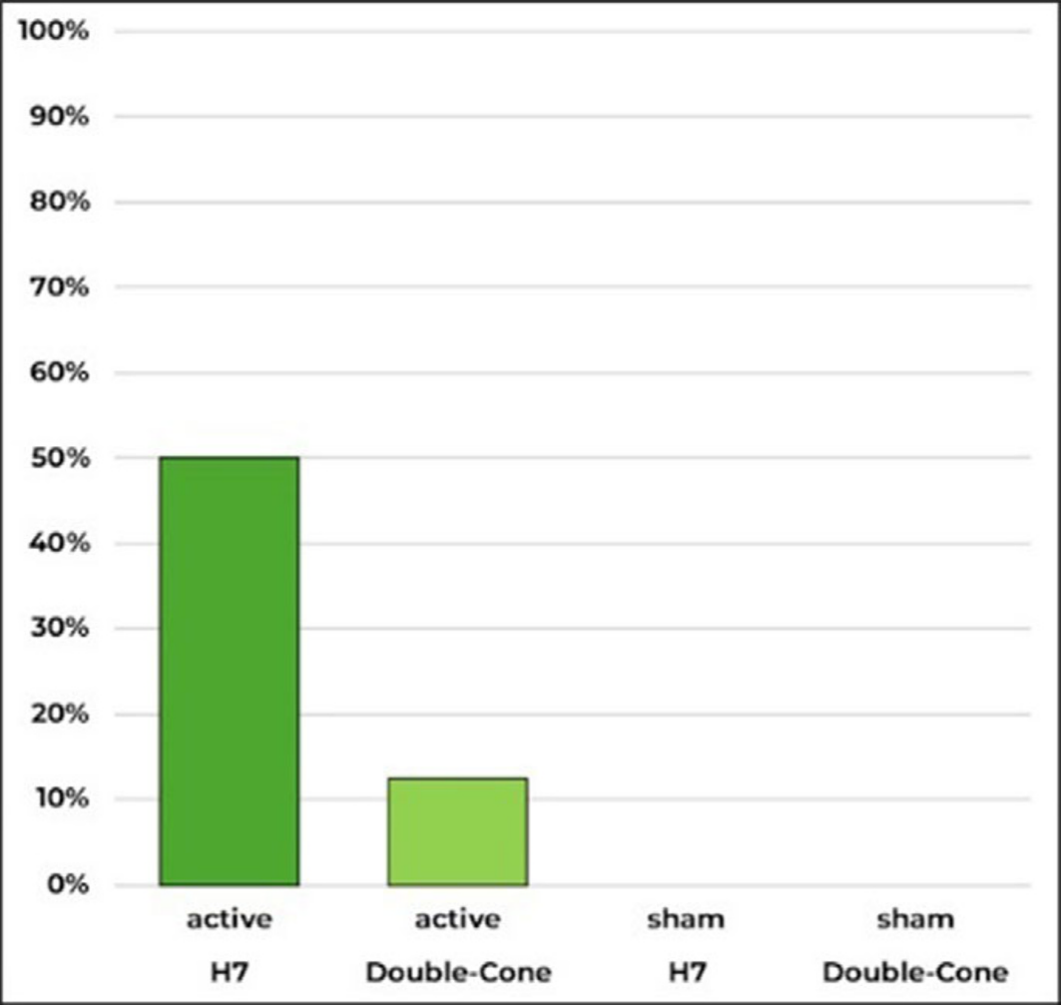

**Image 3:**
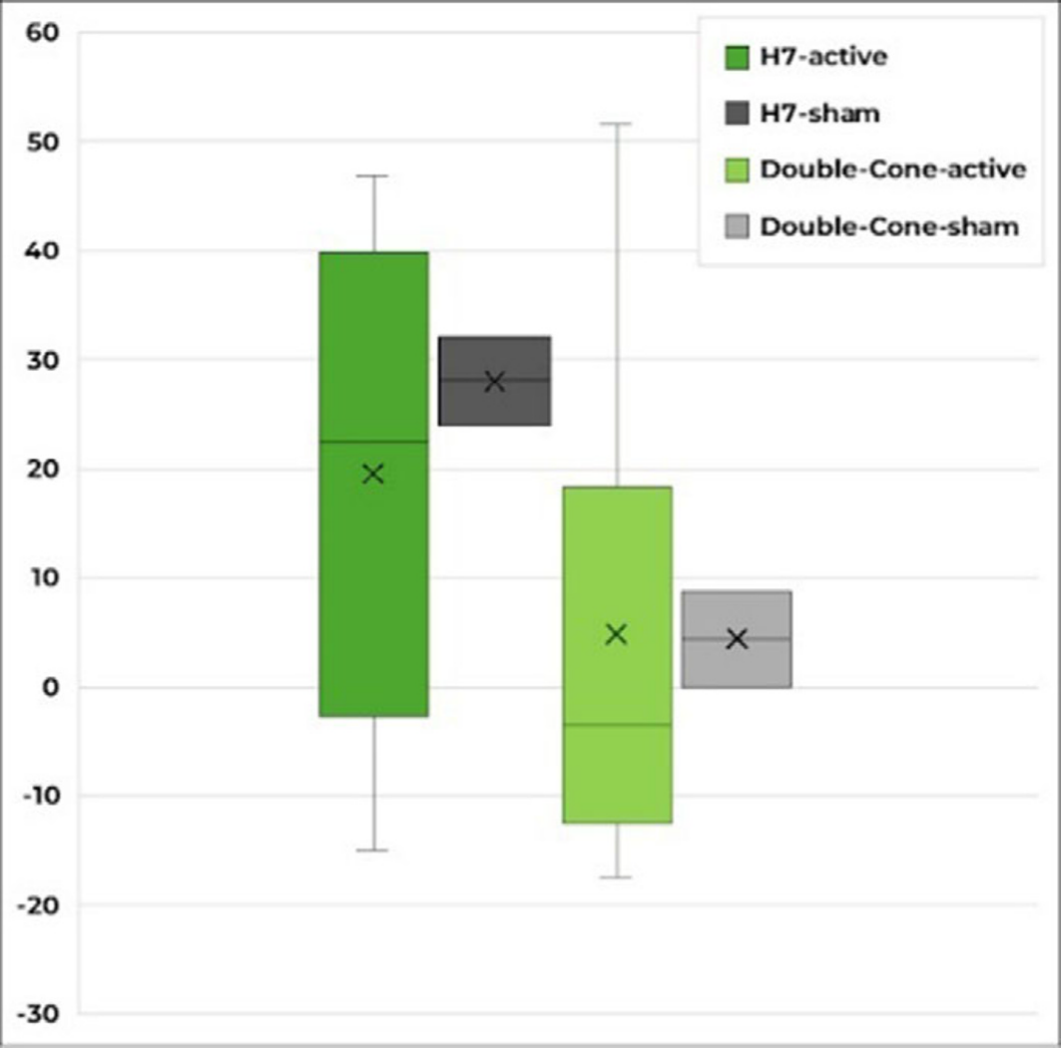
Image 3: Long description.

**Conclusions:**

Although the difference between the coils didn’t reach significance, owing to the small sample size, our data suggest that the **H7** coil **may be more effective** than the **Double-Cone** coil for **OCD**, and that **shorter illness duration** is associated with a **higher likelihood of response**.

**Disclosure of Interest:**

M. Jakubec: None Declared, O. Laskov: None Declared, N.-A. Böhmová: None Declared, N. Biačková: None Declared, A. Čechová: None Declared, T. Novák: None Declared, M. Klírová Grant / Research support from: Received honoraria in the form of travel reimbursement from Brainsway Inc. (Israel) and consulting fees from BTL Industries JSC (Czechia) and Deymed (Czechia).

## Long descriptions

### Image 3: Long description

The Y-axis ranges from negative 30 to 60 in increments of 10. A legend in the top right identifies four categories: H 7-active (dark green), H 7-sham (dark gray), Double-Cone-active (light green), and Double-Cone-sham (light gray).

From left to right:

1. H 7-active: A tall dark green box spanning from approximately negative 3 to 40. The median line is at 23, and a black X marking the mean is at 20. Whiskers extend down to negative 15 and up to 47.

2. H 7-sham: A short dark gray box positioned to the right of the first box, spanning from 24 to 32. The median line and mean X are both at 28. There are no visible whiskers.

3. Double-Cone-active: A light green box spanning from negative 13 to 18. The median line is at negative 3, and the mean X is at 5. Whiskers extend down to negative 18 and up to 52.

4. Double-Cone-sham: A short light gray box spanning from 0 to 9. The median line and mean X are both at 5. There are no visible whiskers.

Navigate back to Image 3:.

